# Landscape review of active vaccine safety surveillance activities for COVID-19 vaccines globally

**DOI:** 10.1016/j.jvacx.2024.100485

**Published:** 2024-04-10

**Authors:** Parisa A. ShamaeiZadeh, Carmen Villamizar Jaimes, Maria Deloria Knoll, Emmanuelle Espié, Rebecca E. Chandler

**Affiliations:** a International Vaccine Access Center, Johns Hopkins Bloomberg School of Public Health, Baltimore, MD, United States; b University of Kentucky, College of Medicine, Lexington, KY, United States; c Coalition of Epidemic Preparedness Innovations, London, UK; d Coalition of Epidemic Preparedness Innovations, Oslo, Norway

**Keywords:** Vaccine, Safety, COVID-19, Active surveillance system

## Abstract

**Background:**

Evidence of COVID-19 vaccine safety relied upon the global vaccine monitoring infrastructure due to shortened clinical development timelines and emergency use licensure. Differences in AVSS capacity between high-income countries (HICs) versus low- and middle-income countries (LMICs) were known prior to the pandemic.

**Objective:**

To assess the global landscape of COVID-19 vaccine AVSS activities to identify gaps in safety evidence generation across vaccine products and populations with a focus on LMICs.

**Methods:**

A cross-sectional survey was conducted in January 2022 on AVSS activities evaluating adverse events following immunization (AEFI). Data collected included country, targeted population, COVID-19 vaccine product(s), design of surveillance/monitoring activities or study, and AEFIs to be monitored.

To supplement these findings, we conducted a literature review of COVID-19 vaccine safety activities published in PubMed through January 2023. Observational activities assessing AEFI, specifically adverse events of special interest (AESI), following routine use of COVID-19 vaccines in medical practice were included; systematic reviews, benefit/risk assessments, clinical trials, and case reports/series were excluded.

**Results:**

The survey, completed by 34 respondents and compiled with reviews of 7 publicly available Risk Management Plans from five vaccine manufacturers, identified 79 monitoring activities in HICs, 24 in LMICs, and 9 in multiple regions. Most activities in LMICs were planned cohort event monitoring (CEM) studies (n = 18); two multi-national hospital-based sentinel surveillance studies for AESI were ongoing. Activities in LMICs evaluated multiple COVID-19 vaccine products simultaneously and were sponsored by health authorities. The literature review identified 1245 unique citations, of which 379 met inclusion criteria. The majority evaluated vaccines primarily used in high-income countries: Pfizer BioNTech (Comirnaty; n = 303), Moderna (mRNA-1273; n = 164), AstraZeneca (AZD1222; n = 126), and Janssen (Ad26.COV2.S); n = 62); 14 citations assessed vaccines used exclusively in LMICs: Sinovac (CoronaVac), Beijing CNBG (BBIBP-Corv), Bharat (Covaxin), SII (Covashield), and Gamaleya (Gam-Covid-Vac) vaccines.

**Conclusions:**

Robust safety evidence for input into benefit/risk assessments is likely unavailable for most COVID-19 vaccines used primarily in LMICs due to emphasis on cohort event monitoring methods. Goals for equitable vaccine access should be coupled with investment and support for building infrastructure and capacity for safety evidence generation to inform policy and regulatory decisions at local levels.

## Highlights

• Although all regions had safety activities, few publications exclusively in LMICs were found.
• Our survey identified many unpublished safety activities in LMICs, suggesting that evidence of vaccine safety in published literature is incomplete.
• Further investment will be required to advance AVSS capacities in LMICs to estimate incidence of serious AEFIs and any attributable risks from vaccination.

## Introduction

The World Health Organization (WHO) announced COVID-19, caused by the SARS-CoV-2 virus, as a global pandemic on March 11, 2020 [1]. This announcement came amidst a global crisis given the heightened severity of and population susceptibility to the novel disease, leading to an unprecedented goal of developing vaccines rapidly, which was demonstrated by the United States’ enactment of “Operation Warp Speed” [2]. As a result of these efforts, 12 billion doses COVID-19 vaccines have been rolled out worldwide and have been estimated to have saved 20 million lives [1].

Emergency-use authorization of some COVID-19 vaccines relied heavily on Phase 3 clinical trial safety and efficacy data. However, even large trials evaluating thousands of individuals are too small to provide robust estimates of all potential risks with large-scale use in populations. Assessing safety in real-world use conditions is critical to fully characterize the safety profile of a vaccine, including events occurring at rare frequencies with longer time to onset, or unique to or in excess rates in groups excluded or underrepresented in clinical trials, such as pregnant or immunocompromised persons, people with chronic illnesses or comorbidities, and populations from different genetic backgrounds [3]. Fully estimating the risks associated with vaccination as we do with the benefits of vaccine efficacy and effectiveness is critical to assessing the benefit-to-risk ratio necessary to maintain public confidence and inform choice [4].

Safety surveillance for serious AEFI typically requires two complementary systems, passive and active. Passive surveillance systems can be used for generating hypotheses of new causal associations between rare AEFI and vaccines. These systems rely upon astute health care providers and patients for the generation of “spontaneous” reports of AEFI, pooling these observations over populations larger and more diverse than included in clinical trials [5]. Limitations of passive surveillance systems include underreporting, unknown denominators necessary to estimate incidence, and lack of appropriate comparator groups. Active vaccine surveillance systems (AVSS) allow for comparison of incidence of AEFI between vaccinated and unvaccinated populations. AVSS activities can take various forms, largely based upon available resources. Cohort event monitoring (CEM) is a common design that aims to capture all adverse effects by following patient cohorts after receiving a pharmaceutical product. Sentinel site surveillance is a more targeted approach that recruits multiple health facilities as part of a “network” to report adverse effects that present at their facilities; the most resource intensive but most robust study design, AVSS, requires formal epidemiological studies via electronic healthcare databases [6].

The aim of this project was to perform a landscape review of global AVSS activities evaluating safety of COVID-19 vaccines to ascertain gaps related to specific vaccines, geographic regions, or specific subpopulations evaluated, focusing on LMICs.

## Methods

We conducted a global cross-sectional survey to collect information from individuals working on safety monitoring activities. Although this survey found activities not published in the literature, it was evident that not all safety activities were captured. To collect a more complete landscape of COVID-19 vaccine safety activities, we also conducted a literature search of published safety monitoring activities to supplement responses from the survey. Therefore, our methods aimed to capture activities published in the literature as well as activities by investigators who disclosed their activities via survey.

### Survey:

The survey, conducted from December 2021 to February 2022, assessed planned and ongoing AVSS activities or targeted observational activities evaluating AEFI in settings of routine COVID-19 vaccine use (i.e., clinical trials were excluded). The survey was sent out by members of the COVAX Vaccine Safety Working Group with representation from the International Federation of Pharmaceutical Manufacturers and Traders, Developing Countries Manufacturing Network, the Brighton Collaboration, CEPI and the WHO to individuals at numerous institutions worldwide, including WHO regional offices, universities known to be evaluating COVID-19 vaccines, government public health institutions, and vaccine manufacturers. Information collected included the surveillance or study site location, design, vaccine(s) evaluated, population(s) evaluated, and types of adverse event(s) evaluated [Supplemental Annex 1]. Outcome categories included any adverse event (i.e. any adverse response post vaccination), serious adverse events (i.e. any adverse event requiring medical attention), or specific adverse events (i.e. monitoring of predetermined adverse events).

Vaccine manufacturers were also asked to provide Risk Management Plans (RMPs) for their COVID-19 vaccines with Emergency Use Authorization, which describe post-licensure commitments for specific planned safety activities (i.e., post-introduction observational studies) in countries where their vaccines were in use.

### Literature review:

The search strategy, developed with assistance from Johns Hopkins Library Sciences in October 2022, identified English-language articles in PubMed published between January 2021 and January 2023 [Supplemental Annex 2]. Eligible activities included papers that met the following criteria: observational or non-interventional studies evaluating serious or specific adverse events; assessed causality or association with the investigational product and collected data through an active surveillance system. Reactogenicity studies, cross-sectional surveys, clinical trial data, and case series/reports were excluded. Additionally, data from passively collected systems such as VAERS in the US and VigiBase were excluded due to inability to determine attributable risk from passive surveillance data. Data on the country, vaccine, type of study, target population, sample size, adverse events evaluated, and key results presented in the abstract were abstracted from included articles into REDCap. For our literature search, we defined AESIs as those listed by the Safety Platform for Emergency Vaccines [7].

Countries were categorized into income groups using The World Bank classification [8]. Activities involving multiple countries were listed as “unknown country” when unfeasible to tease apart responses with multiple components.

A module was developed on the publicly available website https://www.VIEW-hub.org to house the results of the survey and literature review abstraction, which is searchable by vaccine, region, adverse event, and population.

## Results

**Fig. 1:**
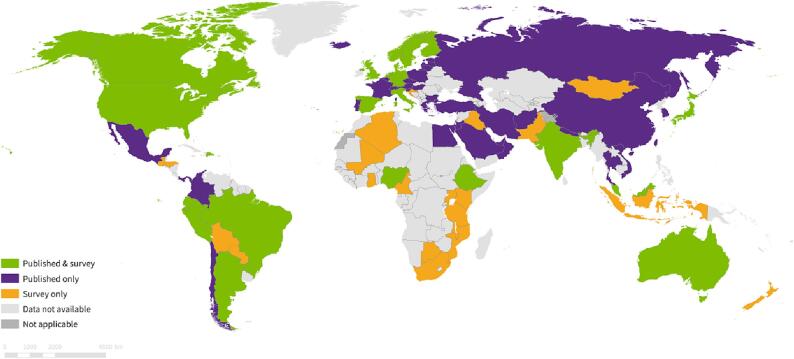
Mapping of COVID-19 vaccine safety studies by method Identified (survey and published literature).

Overall, 70 countries were identified via literature review and survey methods. The three predominant types of monitoring activities identified were Cohort Event Monitoring (n = 186), Prospective Cohort (n = 74), and Self Control Case Series (n = 64). Although activities were reported in all geographic regions, Europe had the most activities (n = 190), followed by the Americas (n = 149) and the Western Pacific (n = 96). Most activities were conducted in high income countries (n = 363) with the least amount of activities in low-income countries (n = 13) [Fig. 1, Table 1].

**Table 1:** Characteristics of activities identified by variable.

**Region (WHO Classification 2023)**	**Literature Review**	Survey*	**Total**

Europe	161	29	190
Americas	109	40	149
Western Pacific	89	7	96
Multiple regions	32	9	41
South-East Asian	21	4	25
Eastern Mediterranean	17	2	19
African	2	21	23

**Income Strata (World Bank 2023**)	**Literature Review**	Survey*	**Total**

High income	301	62	363
Upper middle income	70	17	87
Lower middle income	26	13	39
Low income	2	11	13

**Activity Design**	**Literature Review**	Survey*	**Total**

Cohort-event monitoring	124	62	186
Prospective cohort study	74	0	74
Case-cohort	55	6	61
Retrospective cohort study	54	0	54
Case-control	36	26	62
Self-control case series	41	23	64
Sentinel site surveillance	19	32	51
Enhanced/targeted/spontaneous reporting	17	27	44
Cohort study	15	0	15
Other	11	0	11

**Vaccine**	**Literature Review**	Survey*	**Total**

Pfizer BioNTech (Comirnaty)	312	40	352
Moderna (mRNA-1273)	172	27	199
AstraZeneca (AZD1222)	131	35	166
Janssen (Ad26.COV 2.S)	68	25	93
Sinovac (CoronaVac)	43	6	49
Beijing CNBG (BBIBP-CorV)	16	7	23
SII (Covishield)	11	7	18
Bharat (Covaxin)	11	0	0
Multiple/Unspecified	10	28	38
Gamaleya (Gam-Covid-Vac)	9	3	12
Gamaleya (Sputnik Light)	5	0	5
Nuvaxovid (NVX-CoV2373)	4	0	4
Novavax (NUVAXOVID)	4	0	4
CanSino (Ad5-nCOV)	4	0	4
Wuhan CNBG (Inactivated)	2	0	2
CIGB (CIGB-66)	1	0	1
Bivalent Moderna (Bivalent mRNA-1273)	1	0	1
Bivalent Pfizer BioNTech (Bivalent Comirnaty)	1	0	1
Finlay (FINLAY-FR)	1	0	1

* The survey structure allowed respondents to select all variables that apply, which led to overlapping numbers. All survey counts are not indicative of unique studies, as some studies may have responded with multiple variables.

### Survey

**Fig. 2:**
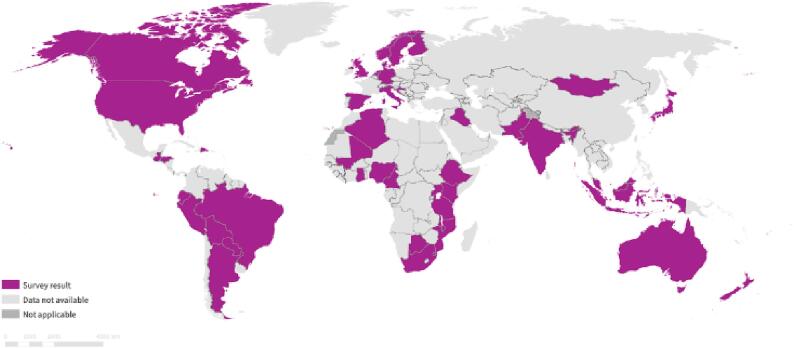
Mapping of COVID-19 vaccine safety activities identified by survey.

A total of 34 survey responses and 7 RMPs provided by five manufacturers (Moderna, Pfizer, Janssen, AstraZeneca, and Novavax) were collected that reported total 112 planned or going activities in 46 countries, with 79 activities located in HIC and 24 activities in LMICs and 9 activities in multiple regions; six respondents reported no vaccine safety activities [Fig. 2]. Vaccines reported being evaluated included Pfizer BioNTech (Comirnaty) vaccine (n = 40), Moderna (mRNA-1273) vaccine (n = 27), AstraZeneca (AZD1222) vaccine (n = 35), and Janssen Vaccine (n = 25). 38 respondents noted evaluating multiple vaccines or did not specify in detail which vaccines they were evaluating. Only two multi-country studies using a sentinel site approach for the identification of AESI were reported from the survey, one in Latin America, sponsored by the Pan American Health Organisation and one in Africa, sponsored by Gavi and executed by the Global Vaccine Data Network.

### Literature review

**Fig. 3:**
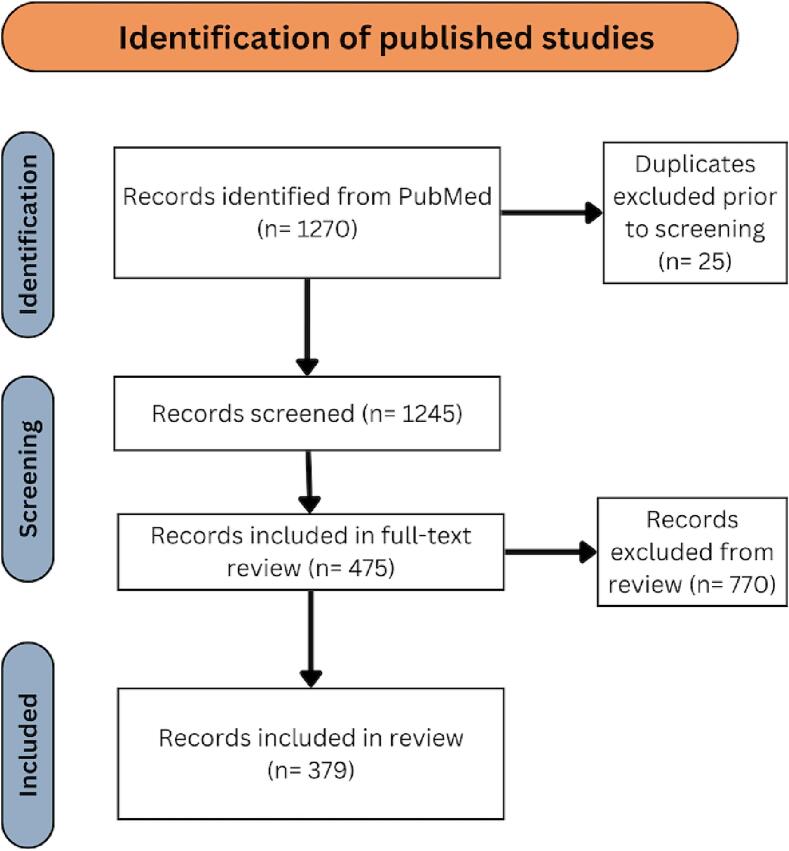
PRISM Chart detailing the review process of relevant published literature.

The literature review identified 1245 unique citations after removing duplicates, which after title and abstract screening for inclusion criteria, resulted in 475 citations eligible for full-text review. A total of 379 unique studies, after excluding studies reported in more than one citation, met criteria and were included in analyses [Fig. 3].

**Fig. 4:**
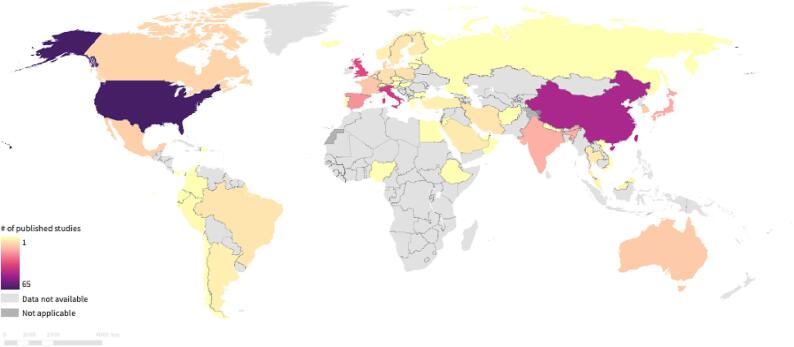
Mapping of COVID-19 vaccine safety studies identified via literature search.

While activities were reported in all major geographic regions encompassing 46 countries, two-thirds of all AVSS activities were conducted in just five countries: United States (n = 60), China (n = 46), Israel (n = 33), Italy (n = 32), and Great Britain (n = 28). Europe had the highest number of activities (n = 161), followed by the Americas (n = 109) and the Western Pacific (n = 89). The three predominant types of activities in published studies were CEM (n = 124), Prospective Cohort (n = 74), and Retrospective Cohort activities (n = 54) [Fig. 4].

The majority of activities identified via literature search evaluated vaccines primarily used in high-income countries, such as the Pfizer BioNTech (Comirnaty) vaccine (n = 312), Moderna (mRNA-1273) vaccine (n = 172), AstraZeneca (AZD1222) vaccine (n = 131), and Janssen Vaccine (n = 68). Assessments for various other vaccines were also identified, including activities evaluating Sinovac (CoronaVac), Beijing CNBG (BBIBP-Corv), Bharat (Covaxin), SII (Covashield), or Gamaleya (Gam-Covid-Vac) vaccines.

## Discussion

This landscape review revealed that the majority of AVSS activities evaluating safety of COVID-19 vaccines have been conducted in HICs and assess only a fraction of vaccines in use. Equitable access to contextually relevant evidence of vaccine safety has not been provided in parallel with access to vaccines, potentially complicating public health efforts relating to vaccine acceptance. This underscores the global dependence on high-income nations for data collection regarding vaccine development and surveillance by inhibiting the self-sufficiency of low and middle-income countries in participating fully in vaccine development and monitoring endeavors.

## Differences in vaccine safety surveillance capacity between high and low income countries

Well appreciated prior to the pandemic was inequity in ability to generate evidence of vaccine safety between high-income countries (HICs) and low- and middle-income countries (LMICs) [9]. Through decades of investment and previous health crises such as the H1N1 influenza pandemic, HICs have created comprehensive systems, most notable in their capacity to detect serious adverse events following immunization (AEFI), estimate attributable risk caused by vaccination, and incorporate identified risks into benefit-risk assessments. National regulatory authorities (NRAs), immunization committees, and vaccine manufacturing authorization holders (MAHs) have established pathways for communication, data sharing, and cooperation, facilitating the identification and closure of any safety evidence gaps and responses to unexpected safety signals [10], [11], [12], [13], [14]. In contrast, infrastructures in LMICs have largely been limited to minimal capacities for vaccine safety surveillance with a priority on the identification of quality issues and programmatic errors [15]. Reports of AEFI are largely collected within national immunization programmed (NIP) with minimal sharing to NRAs [16]. Further, with only weak or absent regulations related to pharmacovigilance in many LMICs, local vaccine manufacturers or MAHs lack mature and robust pharmacovigilance and quality management systems required to perform their own vaccine safety surveillance and implement risk management plans, including post-authorization safety studies, according to international standard [17].

In an effort to address these gaps in vaccine safety surveillance capacity in LMIC and in alignment with the efforts of COVAX to ensure equitable access to COVID-19 vaccines, the WHO produced guidance and tools to increase safety surveillance capacities in LMIC beyond monitoring for programmatic errors and quality issues to the identification and estimation of the incidence of serious AEFI [18]. Key recommendations contained within the COVID-19 vaccine safety surveillance manual related to establishing channels of data exchange between the NIP and NRA and generating evidence of safety through active surveillance of the adverse events of special interest (AESI) for COVID-19 vaccines designated by the Brighton Collaboration within the project, “Safety Platform for Emergency Vaccines” (SPEAC), funded by the Coalition of Epidemic Preparedness Innovations (CEPI) [7]. Additionally, to support the execution of AVSS, a template for a cohort event monitoring (CEM) was provided [19].

There is evidence that the COVID-19 pandemic has stimulated strengthening in passive surveillance systems in LMIC. Improvements were largely noted in the collection of AEFI reports and within data management systems. Increased public awareness and stimulated reporting increased AEFI reporting into passive reporting systems [20], and a tool to facilitate capture of AEFI reports from NIP in the WHO global database was implemented [21]. National regulatory authorities engaged in new regional collaborations for work-sharing and signal management, most notably in Africa’s African Union Smart Safety Surveillance (AU-3S) [22]. Digital innovations were applied, such as mobile phone applications for AEFI spontaneously reported by patients and health care providers [23], as well as data triangulation from multiple sources into a single data management system to support decision making [24]. The consequence of these advancements in the capacity for signal detection by passive surveillance, will be the need for continuous improvement, including investments in sustainable infrastructures for further hypothesis-generating or testing by robust well-designed safety activities.

## The need for more advanced safety surveillance to support the implementation and deployment of new vaccines into LMIC

Building more advanced capacity for the generation of evidence of safety through active vaccine surveillance systems in LMIC has been identified as a key priority by global stakeholders. Direct introduction of new vaccines against infectious diseases in lower resource settings, such as dengue, malaria, Lassa fever requires capacities beyond monitoring for programmatic errors and quality issues; it demands the infrastructure for the generation of evidence to support benefit/risk assessments by both regulators and immunization policy makers. One important lesson from one of the first instances of introduction of a new vaccine directly into LMIC, that of a group A meningococcal vaccine into the Burkina Faso, Niger and Mali in 2010, was the necessity of active surveillance systems for adequate monitoring of serious AEFI [25]. The Global Vaccine Safety Blueprint 2.0, published in 2021, includes an objective to monitor the safety of novel vaccines for emerging infectious diseases, in particular during emergencies, and the strategy to ensure that active vaccine safety surveillance is set-up prior to vaccine introduction [26].

Our survey results identified CEM as the most common approach to AVSS in LMIC, likely a reflection of the guidance and CEM template provided in the WHO COVID-19 vaccine safety surveillance manual. CEM is a form of active surveillance which uses a prospective, observational design to identify the occurrence of adverse events associated with one or more medicines in a single cohort of exposed individuals. Based upon principles established by New Zealand Intensive Medicines Monitoring Programme [27] and the UK Prescription Event Monitoring [28], CEM is primarily considered as a form of observation of a new medicine in routine clinical practice in the early post-marketing phase. CEM has been promoted by the WHO for monitoring new medicines used in public health programmes [29], and there are a number of examples of successful implementations in the published literature [30], [31].

CEM was extensively used to monitor the safety of COVID-19 vaccines in both HIC and LMIC. It is notable that the aims of the use of this design have been variable across publications, from describing and comparing the overall reactogenicity of the different covid-19 vaccines [32], [33], to characterizing safety profiles in subpopulations, such as children and pregnant women [34], [35], and even for “signal detection” [36], [37]. However, the rarity of serious adverse events after vaccination challenges the ability of CEM to identify any pre-specified AESI or unexpected safety signals which typically occur in the range of 1/10,000 to 1/100,000. Only recently a resource-intensive, multi-national CEM conducted by Sanofi on AESI identified for Dengvaxia reported a total of 644 AEFI among 12,594 vaccinated subjects who were contacted 3 times at 6-week intervals and then quarterly for up to 5 years [38].

The lack of electronic records documenting receipt of vaccination and health outcomes in LMIC complicate the execution of formal epidemiological studies using either prospective or retrospective design in LMIC compared to HIC. The challenges faced by covid vaccine developers and manufacturing authorisation holders (MAH) to conduct post-authorisation safety studies in LMIC. In this publication, pharmacovigilance experts from industry call for a collective strategy to build LMIC sentinel sites [39]. This challenge of MAH was reflected in the results from our survey, as the only studies using a sentinel site approach for the identification of AESI were executed by public health organisations.

Our work contributes to other published literature characterizing the global landscape of active vaccine safety surveillance activities during the COVID-19 pandemic response. A recently published systematic review identified all peer-reviewed observational COVID-19 vaccine safety monitoring activities in LMICs [40]. Fifty-eight activities were included in the analysis. A quarter of the activities used a cohort study design, and the remaining were cross-sectional activities, and the median sample size of the activities was less than 500 subjects. In half of the activities, vaccination data relied upon self-reporting of the study participant. The recommendations of the authors were that efforts are needed for improving the quality and rigor of AVSS in LMIC settings. Further investigation is needed to confirm findings and to identify potential limitations in safety data collection, infrastructure, and availability. This is particularly crucial given that many of these countries are low and middle-income nations heavily reliant on safety systems. They are also key recipients of vaccine introduction assistance from international organizations. Available tools and guidance to support active vaccine safety surveillance in LMIC.

To guide further progress beyond CEM, a roadmap for international collaboration for epidemiologic monitoring of safety and effectiveness of new high priority vaccines is available [41]. The recommendation includes 1) integrated, coordinated passive surveillance, including a consideration for stimulated, active reporting of a small number of relevant AESIs, and 2) international collaborative epidemiological studies, consisting of two complementary approaches, a) monitoring of effectiveness and the more common, nonserious AEFI within a consortia of health and demographic sites and 2) monitoring of less common, serious AEFI within a network of sentinel hospitals, using a self-controlled case series design. Open access to standardized case definitions of AESI, including companion guides for their use, is provided by the Brighton Collaboration at their website. The feasibility of an international hospital-based network for the assessment of potential epidemiological associations between AESI and vaccines in any setting, including LMIC, has been demonstrated in a proof-of-concept study published in 2018 [42]. and operational lessons learned have been shared [43].

To support country level organizations responsible for the implementation of new vaccination programs in resource-limited countries, a CIOMS Guide to Active Vaccine Safety Surveillance was published in 2017 [44]. It describes key background concepts, a process for evaluating whether significant knowledge gaps exist and if passive safety surveillance is adequate, as well as providing methodical approaches for conducting active surveillance using either individual- or aggregate-level data. The CIOMS Guide also includes practical aspects of execution of AVSS.

A recent addition to the growing pool of resources for building capacity in vaccine surveillance are tools to support benefit/risk assessments. Guidance on Benefit-Risk Balance for Medicinal Products is awaited from CIOMS describing quantitative and qualitative approaches to the evaluation of benefit-risk, including visualization of benefits and risks to improve transparency and understanding amongst key stakeholders [45]. More specific for vaccines is the “Brighton Collaboration Standardized Module for Vaccine Benefit-Risk Assessment” [46].

## Limitations of our assessment

This study was performed to map the activity of global AVSS, and as such, there are numerous limitations to the conclusions that can be drawn from the results. The survey was distributed electronically through networks of vaccine safety experts associated with academic, industry as well as local and regional regulatory and public health agencies, and it is thus difficult to make precise estimates of the response rate. Further, the responses received only represent those who voluntarily shared their study details (or, for RMPs, were publicly available) and are not comprehensive of all planned and ongoing vaccine safety activities for COVID-19 vaccines worldwide. Although our survey provided insight to some AVSS activities, the authors decided to not systematically follow-up with survey responses for activity updates (e.g. if the activity was still ongoing, published, or halted). Rather than systematic follow-up of all survey responses, the authors decided to conduct the supplemental literature review. Neither information beyond the overall design of the study nor a detailed description of study population was collected in our survey, limiting the ability of our study to conclude on the quality, feasibility or robustness of these AVSS or to confidently match activities that may have been captured in both the survey and in the published literature. Our literature review was limited in time and to articles published in the English language. Further, out of scope of this review was an exploration of challenges and obstacles to the performance of AVSS in LMIC.

This assessment of AVSS during the global COVID-19 immunization campaign demonstrates a continued reliance of LMIC on HIC for the generation of evidence of vaccine safety. These results suggest that infrastructures and resources are currently insufficient for the optimal, contextual assessment of benefit/risk during new vaccine introduction in LMIC. Reliance on HIC for generation of evidence will not be possible for early vaccine deployment against endemic, neglected pathogens and for those with epidemic and pandemic potential arising in LMIC. Expansion of existing infrastructures from the identification of programmatic errors to include systems for incidence measures of benefits and serious harms is required. Essential to this evolution is increased collaboration across country stakeholders, including active surveillance systems, to ensure safe distribution and administration of new vaccines through global health disease control efforts.

## Conclusion

Our study identified that most safety data was generated in high income countries for mainly mNRA vaccines. Of activities identified in LMICs, the majority were CEM activities targeting all licensed vaccines in all eligible populations. This assessment brings to light the inequity of monitoring capacity for vaccine safety being used in low- and middle-income countries compared to high income countries. These findings can prompt public health policy interest to prioritize resources for global capacity building around vaccines and vaccination in low- and middle-income countries with limited infrastructure to conduct adequate active vaccine safety surveillance, especially during mass immunization campaigns.

All authors attest they meet the ICMJE criteria for authorship.

## IRB approval status

None required.

## Funding status

Funded by Coalition for Epidemic Preparedness (CEPI).

## CRediT authorship contribution statement

**Parisa A. ShamaeiZadeh:** Writing – review & editing, Writing – original draft, Project administration, Methodology, Investigation, Formal analysis, Data curation. **Carmen Villamizar Jaimes:** Data curation. **Maria Deloria Knoll:** Writing – review & editing, Supervision, Project administration, Methodology, Investigation, Funding acquisition. **Emmanuelle Espié:** Writing – review & editing, Supervision, Resources, Project administration. **Rebecca E. Chandler:** Writing – review & editing, Writing – original draft, Supervision, Resources, Project administration, Methodology, Investigation, Conceptualization.

## Declaration of competing interest

The authors declare the following financial interests/personal relationships which may be considered as potential competing interests: Parisa ShamaeiZadeh reports financial support was provided by Coalition for Epidemic Preparedness Innovations. Parisa ShamaeiZadeh reports a relationship with Coalition for Epidemic Preparedness Innovations that includes: funding grants. If there are other authors, they declare that they have no known competing financial interests or personal relationships that could have appeared to influence the work reported in this paper.

## Data Availability

Data will be made available on request.
