# Supplementary material for: Landscape review of active vaccine safety surveillance activities for COVID-19 vaccines globally
Source: Vaccine X. 2024 Apr 10;18:100485. doi: 10.1016/j.jvacx.2024.100485 (PMC11035105; doi:10.1016/j.jvacx.2024.100485)
Supplement: Supplementary Data 1 [file mmc1.docx]

## Supplementary Appendix 1: Survey Form

**Landscape analysis on Active Vaccine Safety Surveillance (AVSS) of**

**COVID-19 vaccines**

Conducted by the Coalition for Epidemic Preparedness Innovations (CEPI)

(with assistance from The International Vaccine Access Center (IVAC) at the Johns Hopkins Bloomberg School of Public Health)

**Questionnaire – version November 3, 2021**

**Objective:** To better understand the current planning, the status of global, national, or regional governmental and non-governmental activities on active vaccine safety surveillance (AVSS) of COVID-19 vaccines post-authorization, complementing passive pharmacovigilance. This will help WHO and other stakeholders to identify gaps in Vaccine safety evidence data for informing global vaccine policy.

Data may be shared with stakeholders, including policy makers, funders, and study implementers. Some data, such as the location of studies and type of study proposed, might be made publicly available on a public website in the future. If this is a concern, please note that at the end of the questionnaire.

**Instructions:** Please answer the below questions for all AVSS activities which are at least at the stage where there are concrete plans (e.g., budget and/or protocol) for an AVSS. As progress is made on the studies, please provide updates via email response. This form will allow you to fill out information on one AVSS. If you are submitting information on more than one AVSS, please submit separate forms.

| 1. Email Address |
| --- |
| 1. Name of the institution |
| 1. Name of the Respondent |
| 1. Have you secured funding for this AVSS?  - If yes, please list funding source |
| 1. Study name or number |
| 1. Vaccines being evaluated |
| 1. Country or countries the vaccine safety study is conducted in (Choose all that apply.) |
| 1. Safety risks under evaluation*  - Any AEFI - Serious AEFI - Specific AEFI – please describe |
|  |
|  |
|  |

| 1. Study setting (Choose all that apply.) |
| --- |
| - - Hospital / Clinic records |
| - - Multicenter site |
| - - Self- reporting / questionnaires |
| - - Population-based registries / Health and demographic surveillance sites |
| - - Healthcare databases / Large linked databases |
| - - Other – please describe |
| 1. Population being investigated (Choose all that apply.) |
| - Healthcare workers |
| - General adult population |
| - Elderly >= 65 years |
| - Persons with comorbidities |
| - HIV infected |
| - Other immunocompromised (e.g., transplant patients, autoimmune or inflammatory disorders) |
| - Children |
| - Pregnant/breastfeeding women |
| - Others – please describe |
| 1. Study design methodology being used (Choose all that apply.) |
| - - Cohort-event monitoring (CEM) |
| - - Sentinel site surveillance |
| - - Case-control |
| - - Case-cohort |
| - - Self-controlled case series |
| - - Enhanced / targeted reporting/spontaneous reporting |
| - - Clinical trial |
| - - Other – please describe |
| 1. What stage is your study currently? (Choose all that apply.) |
| - Fundraising |
| - Protocol developed |
| - Undergoing ethical review |
| - Data collection |
| - Analysis/writing |
| - Completed |
| - Published/reported externally - Not Applicable |
| 1. Enrollment start date (Month/Year)  *(record planned start date if not begun)* |
| 1. When do you expect to have the results? (Month/Year) |
| 1. Summary of the AVSS approach or project: |
| 1. Any additional comments or details about the study? |
| 1. Is this study mentioned in an RMP document? If so, |
| - - - - 1. Please provide the title or a description |
| - - - - 1. Please provide a link. |
| 1. Is a description of this study publicly available? |
| - - - - 1. If yes, please provide a link |
| - - - - 1. If no, please indicate where information can be found |
| 1. Are results publicly available? If so, |
| - - - - 1. Please provide a link  1. Further questions, comments, or concerns of data availability |
|  |

*These were not defined in the survey but WHO [defined](https://www.who.int/groups/global-advisory-committee-on-vaccine-safety/topics/aefi/serious-aefi) adverse events following immunization (AEFI) as any untoward medical occurrence following immunization which does not necessarily have a causal relationship to the vaccine. The adverse event may be any unfavorable or unintended sign, abnormal laboratory finding, symptom or disease. Serious AEFIs are defined as all-cause deaths and hospitalizations. Specific AEFIs indicate selected AEs are being investigated, such as by a search for specific terms in a database or clinical record review.
