# Supplementary material for: Landscape review of active vaccine safety surveillance activities for COVID-19 vaccines globally
Source: Vaccine X. 2024 Apr 10;18:100485. doi: 10.1016/j.jvacx.2024.100485 (PMC11035105; doi:10.1016/j.jvacx.2024.100485)
Supplement: Supplementary Data 2 [file mmc2.docx]

## Supplementary Appendix 2: Literature Search Terms

PubMed:

(( ("Severe Acute Respiratory Syndrome"[mesh] OR “SARS” [tw] OR “SARS-CoV-2” [tw]) AND “vaccin*"[tw] OR ("COVID-19 Vaccines"[mesh] OR "COVID 19 Vaccin*"[tw] OR "COVID19 Vaccin*"[tw] OR "COVID-19 Vaccin*"[tw] OR "SARS-CoV-2 Vaccin*"[tw] OR "Coronavirus Disease 2019 Vaccin*"[tw] ) )

AND

( ("adverse effect*"[ti] OR "adverse event*"[ti] OR "safety"[ti] OR "side effect*"[ti]) OR ("Myocarditis" [tw] OR "pericarditis" [tw] OR "Guillain-Barre Syndrome"[mesh] OR “Acute Inflammatory Polyneuropath*” [tw] OR "Guillain-barre syndrome"[tw] OR "acute autoimmune neuropath*"[tw] OR " Acute Infectious Polyneuritis"[tw] OR "Facial Paralysis"[Mesh] OR “Facial Pals*” [tw] OR "facial paralys*"[tw] OR “Encephalomyelitis, Acute Disseminated”[mesh] OR “Post-Vaccinal Encephalitis” [tw] OR "Thrombocytopenia"[Mesh] OR “Thrombopenia” [tw] OR "Anosmia"[Mesh] OR “ Smell Loss” [tw] OR "loss of smell"[tw] OR "Ageusia"[mesh] OR “ Taste Loss” [tw] OR "loss of taste" [tw] OR “Anaphylaxis” [mesh] OR “anaphylactic Shock” [tw] OR “ Anaphylactic Reaction” [tw] OR “ Blood Coagulation Disorders” [mesh] OR "Blood Coagulation Disorder*"[tw] OR “Seizures” [mesh] OR “Convulsion” [tw] OR "seizure*"[tw] OR "Petit Mal"[tw] OR "grand mal"[tw] OR “vaccine associated enhanced disease”[tw] OR “acute cardiovascular injury”[tw]) )

AND

( ("observational" [tiab] OR "follow-up study" [tiab] OR "prospective*" [tw] OR "longitudinal" ) OR "study"[tw] OR "rates"[tw] OR "incidence"[tw] OR ("product surveillance, postmarketing"[Mesh] OR "post-marketing"[tw] OR "postmarketing"[tw] ) ))

NOT hesitancy [ti] NOT attitude[ti]

NOT "cross-sectional"[tw] NOT ("case*"[ti] AND "report"[ti]) NOT "predict*"[ti] NOT "case study"[ti]

NOT "review"[publication type] NOT "Systematic Review"[publication type] NOT "Systematic Review"[tw]

NOT "Clinical Trial" [Publication Type] NOT "Controlled Clinical Trial" [Publication Type] NOT "trial" [tw]
